# Development and Optimization of Peanut-Based Beverages: A Malawian Consumer-Driven Approach

**DOI:** 10.3390/foods11030267

**Published:** 2022-01-20

**Authors:** Aggrey Pemba Gama, Koushik Adhikari

**Affiliations:** 1 Department of Food Science and Technology, Lilongwe University of Agriculture and Natural Resources, Lilongwe P.O. Box 219, Malawi; agama@luanar.ac.mw; 2 Department of Food Science and Technology, The University of Georgia, 1109 Experiment St, Griffin, GA 30223, USA

**Keywords:** peanut beverage, optimization, mixture design, product matching, CATA, consumer acceptability

## Abstract

Development studies of peanut-based beverages have been ongoing for many years, but there are still challenges, especially with their sensory properties and, ultimately, consumer acceptability. As a result, peanut-based beverages are rarely found on the market, even in developed countries. The current study used mixture design and product matching approaches to develop and optimize peanut-based beverages. Sensory drivers of consumer acceptability were also determined. Optimization focused on maximizing overall consumer acceptability by varying two independent variables that constituted 16% of the beverage by weight: peanut paste (PP) and malted milk powder (MMP). The optimal proportions of the PP and any type of MMP, in the two-component mixture, were 0.6 and 0.4, respectively. Maintaining all other factors as constant, model validation results showed that the model could predict overall liking of the peanut-based beverages with 96% accuracy when the proportions of PP and MMP are known. The samples that were perceived, by the consumers, to be thick, creamy, and smooth had significantly higher (*p* ≤ 0.05) overall liking scores than those that were perceived to be watery, grainy, and whitish. Based on the findings, acceptable peanut-based beverages were developed and can be scaled up despite using non-defatted peanuts in the formulation.

## 1. Introduction

Malnutrition and non-communicable diseases such as cancer and cardiovascular diseases remain a global challenge [1]. Governments worldwide have devised strategies to promote health by nudging people to consume more nutritious foods and maintain a healthy lifestyle to address this challenge. Among such nutritious foods are legumes, such as peanuts. Unfortunately, food choices are not influenced by anticipated health benefits only, but also the sensory appeal of food, among other factors [2,3,4,5]. Therefore, the development of food products with acceptable sensory properties helps promote consumption. For instance, the development of peanut butter significantly improved consumption of peanuts in the U.S. Peanut butter currently accounts for about half of the edible form of peanuts in the country [6]. Despite the limitation of peanut allergies and sensitivities, globally, peanuts are highly consumed in various forms.

Worldwide, peanuts are primarily consumed in solid (snacks) or semi-solid form (pastes) and rarely as a liquid in the form of a beverage. Compared to solid and semi-solid foods, beverages are convenient, easy to digest, appeal to all age groups, and can be easily delivered in multiple flavor options. Therefore, peanut-based beverages have a higher potential to promote peanut consumption, especially now that consumers are more interested in beverages with health benefits [7,8]. Considering the competitive advantage of beverages over solid and semi-solid foods, development studies of peanut-based beverages have been ongoing for many years, with notable continual improvements in the resultant products’ physicochemical, nutritional, and sensory characteristics. Peanut-based beverages can be complex colloidal systems that affect the beverage’s sensory properties depending on the ingredients. Previous studies have used defatted peanut flour [9,10,11] or peanut protein isolates [12,13] to overcome some of these challenges in formulating the beverage. However, there are still challenges, especially with the sensory properties and, ultimately, the consumer acceptability of peanut-based beverages. As a result, peanut-based beverages are rarely found on the market, even in most developed countries. It is hypothesized that it is possible to develop an acceptable peanut-based beverage through product optimization techniques even when non-defatted peanuts are used. To develop acceptable products, the consumer’s voice must be heard and incorporated into the product design [9]. Consumers have needs and wants; prospects of success are high if a product satisfies those needs and wants [10]. Therefore, in this study, peanut-based beverages were developed and optimized based on consumers’ preferences.

The process of identifying a combination of ingredients that give the maximum desired response or produces a sensory perception similar to a targeted benchmark is called optimization [11]. When a benchmark or predesignated sensory profile is known, product matching is used. Product matching is a well-known sensory technique used to compare the sensory characteristics of a product, especially after reformulation [12]. In food product development, the ultimate goal is to maximize consumer acceptability, which is determined through affective tests. To identify drivers of consumer acceptability, food products are usually characterized using trained panelists and instrumental techniques [13]. However, other novel methods for sensory characterization of food products use consumers during product development. These methods were developed to speed up the food product development process and reduce costs associated with descriptive and instrumental analyses [14,15]. One of such rapid profiling methods involves using a check-all-that-apply (CATA) question. CATA is one of the most novel, simple, reproducible, and valid options for sensory characterization of various products using consumers [15,16]. Therefore, this study also used CATA to provide more insights into the consumers’ hedonic responses to the different peanut-based beverage formulations.

## 2. Materials and Methods

### 2.1. Sample Preparation

Water, peanut paste (PP), sugar, salt, malted milk powder (MMP), and xanthan gum were used in preparing benchtop batches (5 L each) of the beverages. A combination of MMP and PP constituted 16% of the beverage by weight while the rest of the ingredients contributed 84%. The xanthan gum was donated by TIC Gums Inc. (Belcamp, MD, USA), while salt and sugar were bought from Chipiku Plus Stores in Lilongwe, Malawi. Virginia-type peanuts (ICGV-SM 90704) were obtained from ICRISAT, Malawi, and used to make the PP. The peanuts were medium roasted (Lightness, L = ±50), manually blanched, sorted, and eventually milled into a fine paste using a colloidal mill (Hebei Iron-Lion milling machinery Co., Ltd., Cangzhou, China). Two types of MMP were used depending on the formulation specifications. The first type (BMMP) was a mixture of malted barley powder and non-fat dry milk powder (1:20 mixing ratio), while the other (SMMP) had malted sorghum powder instead of the malted barley powder. The ingredients were mixed and an OMNI GLH-01 homogenizer, set at 28,000 rpm, was used to homogenize the beverage mix for one minute. A steam-jacketed pot was used to pasteurize the beverage mix at 85 °C for 5 min. After hot filling, the samples were cooled, immediately, in an ice bath and then, kept at 4 °C until needed for sensory evaluation tests.

### 2.2. Study Design

#### 2.2.1. Step I: Product Optimization

Based on preliminary beverage preparation trials and a literature review [17,18,19,20,21,22,23], sugar, stabilizer, salt, and water levels were pre-determined and fixed. To determine the optimal levels of peanut paste (PP) and barley malted milk powder (BMMP), a non-constrained two-component mixture design was used. In a mixture design, the response is assumed to depend only on the relative proportions of the ingredients present in the mixture. The total amount is held constant, and the response value changes when the proportions of the components making up the mixture change [24]. Therefore, unlike in factorial designs, the difference in the response is a function of the joint blending property of the ingredients in the mixture. The total concentration of the two components (PP and BMMP) was fixed at 16% of the beverage by weight. Design combinations were generated using Design-Expert software (Version 11.0, Stat-Eas Inc., Minneapolis, MN, USA).

#### 2.2.2. Step II: Prediction Model Validation

The optimal and two other sub-optimal formulations were used to validate the overall liking prediction model generated in step I. The experimentally determined overall liking scores for the samples were statistically compared with their respective predicted values from the mathematical model.

#### 2.2.3. Step III: Product Matching

Four formulations containing SMMP were prepared and compared with the optimal BMMP formulation validated in step II (*Target*). Two types of SMMP were used, and they are herein referred to as SMMP-1 (malted sorghum powder: non-fat dry milk powder = 1:20) and SMMP-2 (malted sorghum powder: non-fat dry milk powder = 1:30). Product matching is a well-known sensory technique used to compare the sensory characteristics of a product, especially after reformulation [12]. The need for reformulation could be necessitated by the change of ingredient suppliers, change of ingredients to meet emerging consumer preferences, or part of a product improvement strategy. In this study, the change of malted milk type from BMMP to SMMP was necessitated by the low cost, availability, and extensive use of sorghum in Sub-Saharan Africa instead of barley [25].

### 2.3. Product Evaluations

Different consumer panels evaluated the products in steps I, II, and III of this study. Therefore, three consumer acceptability tests were conducted, in the sensory laboratory, at Bunda College in Malawi. Each test involved at least 50 consumers divided in three different sessions spanning from 8:30 AM to 11:30 AM. A session lasted for approximately 45-min. In steps I and III, consumers evaluated 5 samples in a session while in step II, only 3 samples were evaluated. Water and unsalted crackers (Bakers crisp crackers, RSA) were used to cleanse the palate before evaluating each sample (~30 mL). The samples were coded with 3-digit random numbers and were served cold (7 °C) to the participants. The serving temperature was maintained throughout the test since the samples were served monadically and direct from the storage refrigerator. The sample presentation order was sequential, following a completely randomized balanced block design. The consumers evaluated all the samples, independently, in standard sensory booths under incandescent light. The average room temperature during the evaluations was 26 °C. Participants were invited to participate in one test only through posters and flyers but, they were selected only if they met the inclusion criteria. Malawian consumers who eat peanut-based products frequently (at least 3 times in a week), aged between 18 and 55, and without any allergies or sensitivities towards soy, peanuts, milk, and wheat participated in this study. In all of the three tests, 55% of the participants were women.

Key attributes (appearance, aroma, flavor, texture) and overall liking were scored using a 9-point hedonic scale (1–dislike extremely; 5–neither like nor dislike; 9–like extremely) in all the three steps. However, for step III, the consumers also described each sample through a check-all-that-apply (CATA) question. The terms in the CATA questions were gathered from previous descriptive analysis studies of similar beverages except that the descriptors were expressed using consumer friendly vocabulary. A total of 16 terms (tasty, tasteless, sweet, bitter, salty, sour, aromatic, roasted peanut flavor, smooth, grainy, creamy, stable, brown color, whitish color, thick, and watery) were included in the CATA question. Protocols for the sensory tests were approved by the University of Georgia’s Institutional Review Board (IRB Approval Number: STUDY00004112).

### 2.4. Statistical Analysis

Regression analysis was used to analyze the data in step I, and the following polynomial equation was fitted:

(1) $$Y=\beta_{1}X_{1}+\beta_{2}X_{2}+\beta_{12}X_{1}X_{2}$$

*Y* is the predicted response (overall liking); *β*_1_, *β*_2_ and *β*_12_ are coefficients for each term, while *X*_1_ and *X*_2_ are the coded proportions of PP and BMMP, respectively. The predicted equation for the response variable and the surface plot was generated using Design-Expert software (Version 11.0, Stat-Eas Inc., Minneapolis, MN, USA). Optimization of the independent variable levels was achieved by desirable maximization of the response factor using a numerical optimization procedure of the Design-Expert software.

For step II, experimentally determined mean overall liking scores were compared with the mathematically predicted values. Model validity was deduced from the calculated coefficients of variation, relative errors, and the goodness of fit (R^2^) between the predicted and actual values.

For step III, one-way analysis of variance (ANOVA) and Tukey’s honest significant difference (HSD) tests were used to assess differences in mean hedonic scores and to do pairwise comparisons of mean scores, respectively. Cochran’s Q test was used to analyze the CATA data followed by McNemar (Bonferroni) method for pairwise comparison of citation frequencies [15]. The effect of the CATA responses on the overall liking scores of the samples was determined through mean impact analysis. Sensory maps were obtained through Correspondence Analysis (CA) and only the significant CATA attributes were used in the analysis. Except for step I, all the statistical analyses were conducted using XLSTAT (ver 19.01; Addinsoft, New York, NY, USA).

## 3. Results

### 3.1. Product Optimization and Validation

**Figure 1:**
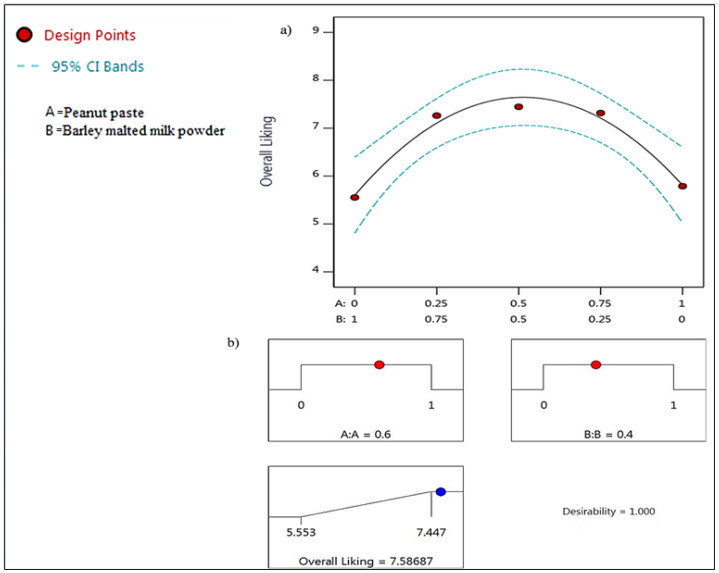
(**a**) Plot showing the effect of levels of the independent variables (peanut paste and barley malted milk powder) on overall liking score of the peanut beverage; (**b**) Graphical representation of the optimized levels of the independent variables. Red dots represent optimal levels of the 2 components (A & B). Blue dot indicates the predicted overall liking score when A & B are at their optimal levels.

Among all the possible models for the response factor (overall acceptability), only a quadratic model was significant (*p* ≤ 0.05), as shown in Table 1. The quadratic response trend is evident in Figure 1a. As a component proportion increased from zero to 1, the overall liking score also increased until the inflection point when the score started to drop. The quadratic model explained 97.7% (Adj. R^2^ = 0.955) of the variation in the overall liking scores of the samples.

Using the numerical optimization (response factor desirable maximization) procedure of the Design-Expert software, the optimal proportions of the PP (*A*) and the BMMP (*B*), in the two-component mixture, were 0.6 and 0.4, respectively (Figure 1b). The prediction equation for the response factor was as follows:

(2) Overall liking score=5.81A+5.60B+7.76AB

The variation between the experimental and predicted values was minimal and none of the coefficients of variation exceeded 5% (Table 2). The model predicted the actual (experimentally determined) values with 96% accuracy (R^2^ = 0.96). Therefore, the mathematical model can predict the overall liking of various formulations of the peanut-based beverages based on PP and BMMP mixture composition.

### 3.2. Product Matching

There were significant differences (*p* ≤ 0.05), among the samples, in all the evaluated parameters except flavor (Table 3). Among the samples (S2-1, S2-2, S2-3, and S2-4) containing sorghum malted milk powder (SMMP), only S2-2 had statistically similar (*p* > 0.05) liking scores as the *Target* (Optimal sample containing barley malted milk powder). Similar to the *Target*, peanut paste and malted milk powder proportions in the two-component mixture were also 0.6 and 0.4, respectively.

**Figure 2:**
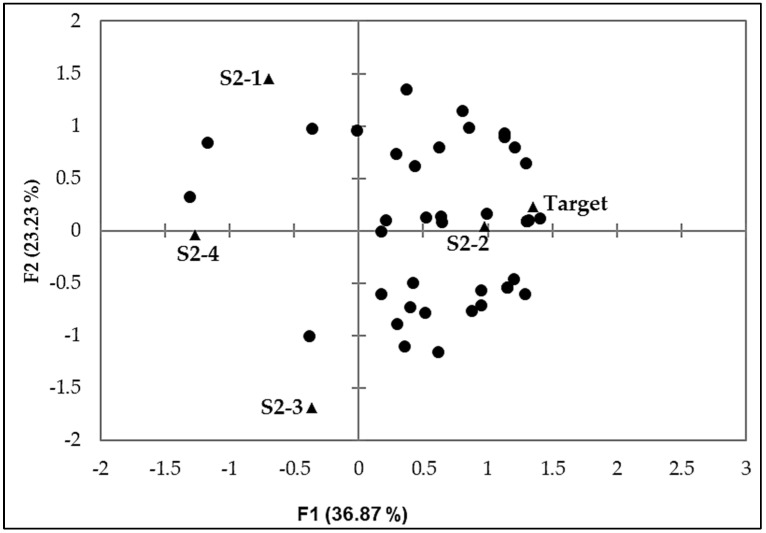
Internal preference map generated using overall liking scores of consumers (●) in product (▲) space.

An internal preference map (Figure 2) was prepared using the overall liking scores of the samples. From the map, it is also evident that only sample S2-2 was closer to the *Target*, and these were the most liked samples as most consumers clustered around them. Therefore, S2-2 was considered the best peanut-based beverage formulation containing SMMP.

The CATA responses revealed differences in the consumer perceptions on the sensory characteristics of the evaluated samples. Significant differences (*p* ≤ 0.05) were found in the citation frequencies of 8 (smooth, grainy, creamy, stable, brown color, whitish color, thick and watery) (Table 4) of the 16 terms that were included in the CATA question. Six terms (thick, smooth, creamy, whitish color, grainy and watery) among the significant eight CATA terms had a significant impact (*p* ≤ 0.05) on the overall mean score. When the terms thick, smooth, and creamy were cited, the overall liking mean score of the sample increased unlike when the terms had not been used. The highest positive mean impact of 0.71 was associated with the term thick, followed by smooth (0.54) and creamy (0.52). On the other hand, when the terms watery, grainy, and whitish color were cited, there was an overall liking mean drop of 0.98, 0.95 and 0.48, respectively. Therefore, the consumers did not like samples perceived to be watery, grainy, and whitish.

**Figure 3:**
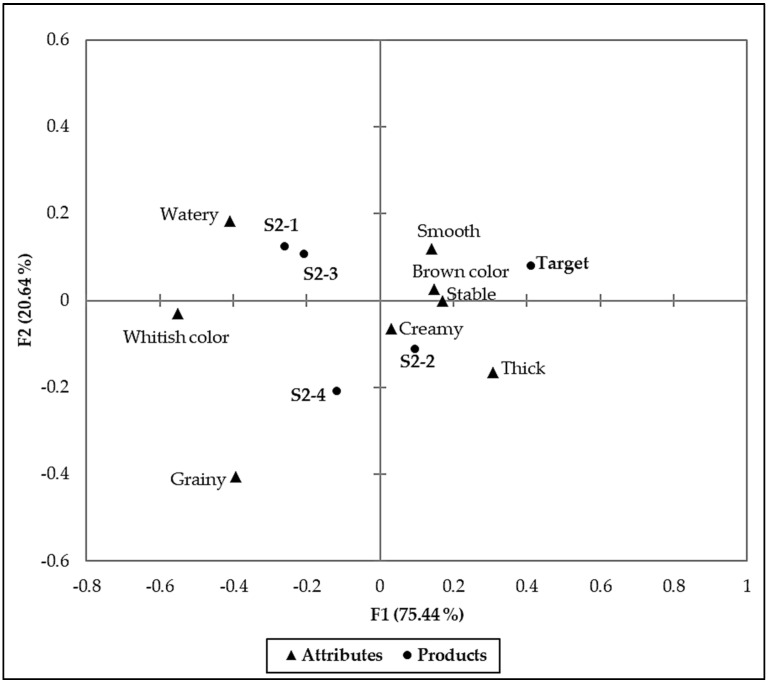
Sensory profile of the beverage samples generated through correspondence analysis (CA) using the significant CATA terms only.

Correspondence analysis (CA) results (Figure 3), using the significant CATA terms only, confirmed the similarity of sensory profiles of sample S2-2 and the *Target*. These samples were associated with terms that had a positive mean impact; therefore, it is not surprising that they had significantly higher overall acceptability scores. S2-2 and the *Target* were located on the positive side of the first dimension on the sensory map and primarily described as smooth, creamy, stable, thick, and brown. The rest of the samples (S2-1, S2-3 and S2-4) were located on the negative side of the first dimension. They were mainly described as watery, grainy and whitish, which had a negative mean impact on overall acceptability.

## 4. Discussion

The quadratic trend in the overall liking of the beverage as the peanut paste proportion increased in the mixture displays a typical consumer response pattern. Consumer acceptability does not follow a linear trend even for generally good attributes such as sweetness. Beyond a certain optimal level, the sweetness is considered to be unappealing, and consumer liking starts to decline. Having more peanut paste in the beverage would likely guarantee more health benefits related to peanut consumption. However, the study has confirmed through the model validation that going beyond a peanut paste proportion of 0.6 in the two-component mixture would compromise consumer acceptability of the beverage. Although health is one of the food choice motives, sensory appeal usually dominates. Eating is, to a greater extent, a source of pleasure and comfort [26]. For instance, despite the health benefits, the global consumption of vegetables is generally low [27]. Many studies have partly attributed the low consumption to most vegetables’ lack of sensory appeal. Therefore, it is important to develop nutritious foods that have acceptable sensory properties as well.

In this study, product matching was an effective shortcut for developing an acceptable peanut-based beverage with SMMP in its formulation. Unlike barley, sorghum is commonly grown in Sub-Saharan Africa, and it is already used to produce both alcoholic and non-alcoholic drinks, among others [25]. Therefore, its use in the peanut-based beverage formulations would make commercialization more feasible, especially since the sensory properties were also acceptable. Raw-beany flavor is an undesirable sensory property that affects the acceptability of beverages from legumes. Therefore, the use of roasted peanuts in this study was of great benefit. Roasting suppressed the raw-beany flavor by enhancing the roasted peanutty flavor and imparting a brown color to the beverage. This had a significant positive mean impact on consumer acceptability. As in other countries, the sensory appeal of food is one of the dominant food choice motives in Malawi [2,3,5,28,29]; therefore, the success prospects of the beverages are high. Gama et al. [3] also found that Malawian consumers prefer more filling foods. Consequently, it is not surprising that beverages perceived to be thick were more liked than those considered to be watery.

Although non-defatted peanuts were used in the formulation, the optimal beverages were perceived to be stable (no phase separation), and this had a positive mean impact on overall acceptability. Peanuts have high oil content, and as a result, the colloidal stability of peanut-based beverages has been a great challenge. Others have used defatted peanut flour [21,22,23] or peanut protein isolates [18,20] to formulate the beverage to overcome this challenge. However, this affects the creaminess of the beverages, an attribute that also had a significant positive mean impact on consumer acceptability in this study. Therefore, the findings of this study confirm the effectiveness of the stabilization system for peanut-based beverages, using non-defatted peanuts, that was developed by Gama et al. [30].

## 5. Conclusions

In the current study, the development of peanut-based beverages with desirable sensory properties was achieved using a two-component mixture design and product matching approaches. Levels of the two key components (peanut paste and malted milk powder), constituting 16% of the total peanut beverage by weight, were optimized to maximize consumer acceptability. The optimal concentrations of the peanut paste and malted milk powder (BMMP or SMMP), in the two-component mixture, were 60% and 40%, respectively. No differences were found between the model predicted overall liking scores and experimental scores. The model equation predicted the actual overall liking scores with 96% accuracy. Therefore, maintaining all other factors as constant, the model can predict the overall liking of various formulations of the peanut-based beverages based on peanut paste and malted milk powder mixture composition. Based on the findings, acceptable peanut-based beverages containing barley malted milk powder and sorghum malted milk powder, respectively, were developed and can be scaled up. However, further studies are required before commercialization. These further studies should focus on market research and impact pathways, packaging and best processing technology, the effect of changing peanut variety, and a shelf-life study when all the key variables have been fixed.

## Figures and Tables

**Table 1:** Model summary statistics.

Source	Sum of Squares	df	Mean Square	F-Value	*p*-Value
Mean vs. Total	222.68	1	222.68	-	-
Linear vs. Mean	0.0276	1	0.0276	0.0245	0.8855
Quadratic vs. Linear	3.29	1	3.29	85.80	0.0115 *
Cubic vs. Quadratic	0.0017	1	0.0017	0.0225	0.9052
Quartic vs. Cubic	0.0750	1	0.0750	-	-
Residual	0.0000	0		-	-
Total	226.08	5	45.22	-	-

* Indicates a significant difference at a 5% significance level.

**Table 2:** Predicted and actual (experimental) overall liking scores for the model validation formulations.

	Mixture Composition		Overall Liking Score			Relative Error (%)
Product	PP	BMMP	Predicted	Actual ^1^	CV (%)	
Optimal	0.60	0.40	7.59	7.53	0.56	0.80
S1-1	0.15	0.85	6.62	6.45	1.29	1.85
S1-2	0.70	0.30	7.38	7.28	0.96	1.37

^1^ Mean value based on responses of consumers; CV = coefficient of variation; PP = peanut paste; BMMP = barley malted milk powder.

**Table 3:** Mean hedonic scores for the samples in comparison to the *Target*.

	Mixture Composition				Mean ± SD				
Product	PP	BMMP	SMMP-1	SMMP-2	Appearance	Aroma	Flavor	Texture	Overall Liking
S2-1	0.6	0.0	0.0	0.4	6.4 ± 1.4 ^b^	6.4 ± 1.1 ^b^	6.4 ± 1.4 ^a^	6.6 ± 1.8 ^b^	6.5 ± 1.4 ^b^
S2-2	0.6	0.0	0.4	0.0	6.7 ± 1.1 ^b^	7.5 ± 1.1 ^a^	7.3 ± 1.3 ^a^	6.7 ± 1.3 ^a,b^	7.7 ± 1.0 ^a^
S2-3	0.5	0.0	0.5	0.0	6.3 ± 1.5 ^b^	6.3 ± 1.2 ^b^	6.7 ± 1.5 ^a^	6.6 ± 1.5 ^b^	6.6 ± 1.5 ^b^
S2-4	0.5	0.0	0.0	0.5	6.1 ± 1.7 ^b^	6.4 ± 1.2 ^b^	6.9 ± 1.6 ^a^	6.5 ± 1.7 ^b^	6.7 ± 1.5 ^b^
*Target*	0.6	0.4	0.0	0.0	8.0 ± 0.9 ^a^	7.3 ± 1.0 ^a^	6.9 ± 1.1 ^a^	7.6 ± 1.1 ^a^	7.5 ± 0.8 ^a^

^a,b^ Different superscripts within each column indicate significant difference (*p* ≤ 0.05); SD = standard deviation; PP = peanut paste; BMMP = barley malted milk powder; SMMP-1 = sorghum malted milk powder type 1 (malted sorghum powder: non-fat dry milk powder = 1:20); SMMP-2 = sorghum malted milk powder type 2 (malted sorghum powder: non-fat dry milk powder = 1:30).

**Table 4:** Cochran’s Q test based on citation frequency of the CATA terms used to describe the samples.

		Citation Frequency (%)				
Descriptor	*p*-Value	S2-1	S2-2	S2-3	S2-4	*Target*
Tasty	0.490	50.0 ^a^	52.6 ^a^	63.2 ^a^	60.5 ^a^	60.5 ^a^
Tasteless	0.483	5.3 ^a^	2.6 ^a^	7.9 ^a^	0.0 ^a^	5.3 ^a^
Sweet	0.690	73.7 ^a^	76.3 ^a^	84.2 ^a^	76.3 ^a^	73.7 ^a^
Bitter	0.406	13.2 ^a^	5.3 ^a^	10.5 ^a^	5.3 ^a^	5.3 ^a^
Salty	0.105	13.2 ^a^	15.8 ^a^	10.5 ^a^	23.7 ^a^	5.3 ^a^
Sour	0.255	0.0 ^a^	5.3 ^a^	0.0 ^a^	2.6 ^a^	0.0 ^a^
Aromatic	0.092	39.5 ^a^	43.2 ^a^	47.4 ^a^	39.5 ^a^	55.3 ^a^
Roasted peanut flavor	0.377	65.8 ^a^	63.2 ^a^	71.1 ^a^	57.9 ^a^	73.7 ^a^
Smooth	0.000	50.0 ^b^	57.9 ^a,b^	57.9 ^a,b^	39.5 ^b^	84.2 ^a^
Grainy	0.001	18.4 ^a,b^	23.7 ^a,b^	21.1 ^a,b^	36.8 ^a^	2.6 ^b^
Creamy	0.046	55.3 ^b^	68.4 ^a^	63.2 ^a^	73.7 ^a^	78.9 ^a^
Stable	0.000	44.7 ^b^	44.7 ^b^	36.8 ^b^	52.6 ^a,b^	78.9 ^a^
Brown color	0.000	50.0 ^b^	68.4 ^ab^	57.9 ^b^	52.6 ^b^	89.5 ^a^
Whitish color	0.000	42.1 ^a^	23.7 ^a,b^	39.5 ^a^	36.8 ^a^	2.6 ^b^
Thick	0.000	18.4 ^b^	44.7 ^a,b^	21.1 ^b^	39.5 ^a,b^	57.9 ^a^
Watery	0.000	60.5 ^a^	26.3 ^b,c^	57.9 ^a,b^	36.8 ^a,b,c^	21.1 ^c^

^a,b,c^ Different superscripts within each row indicate a significant difference (*p* ≤ 0.05).

## Data Availability

The data presented in this article can be accessed from the corresponding author on request.
